# Case report: Varicosity of the communicating vein between the left renal vein and the left ascending lumbar vein mimicking a renal artery aneurysm: Report of an unusual site of varicose veins and a novel hypothesis to explain its association with abdominal pain

**DOI:** 10.4103/0971-3026.76050

**Published:** 2011

**Authors:** Sandeep G Jakhere, Deepak A Yadav, Gorakhnath R Tuplondhe

**Affiliations:** Department of Radiology, B Y L Nair Charitable Hospital and T N Medical College, Mumbai Central, Mumbai, Maharashtra - 400 008, India

**Keywords:** Ascending lumbar vein, communicating vein, renal artery, renal vein, varicosity

## Abstract

A communicating vein between the left renal vein and the left ascending lumbar vein has only rarely been reported in the imaging literature. There are very few reports of varicosity of this communicating vein. Nonetheless, awareness about this communicating vein is of utmost importance for surgeons performing aortoiliac surgeries and nephrectomies as it may pose technical difficulties during surgery or cause life-threatening retroperitoneal hemorrhage. Varicosity of this venous channel may be mistaken for paraaortic lymphadenopathy, adrenal pseudo-mass, or renal artery aneurysm. We report a case of a patient with varicosity of this communicating vein, which mimicked a left renal artery aneurysm. A novel hypothesis is also proposed to explain the relationship with abdominal pain.

## Introduction

Dilatation of venous communications in the retroperitoneum is uncommon, with sparse literature about their imaging appearances. Nonetheless, familiarity with these venous communications is important as they can mimic retroperitoneal lymph nodes,[1] adrenal pseudo-mass,[2] and aneurysms of the renal vasculature. Preoperative awareness of these veins is also important for surgeons who perform aortoiliac surgeries and laparoscopic live donor nephrectomy as these can produce technical difficulties during surgery as well as cause life-threatening retroperitoneal hemorrhage.[3,4]

## Case Report

**Figure 1:**
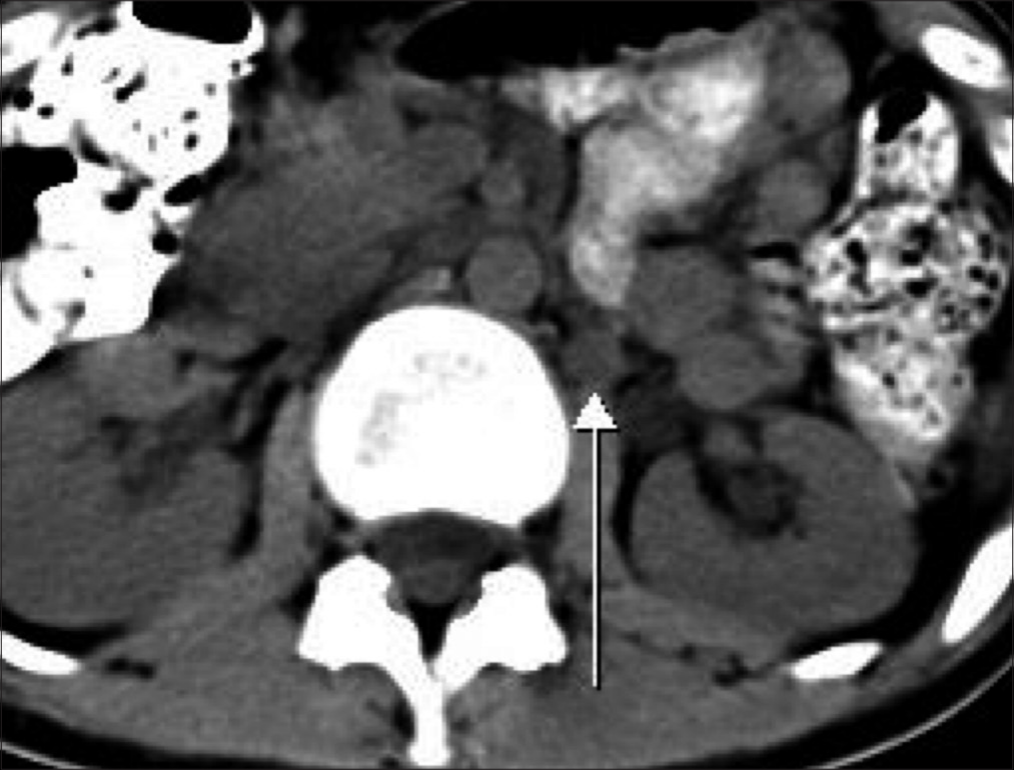
Axial nonenhanced CT scan shows a well-defined round structure (arrow) adjacent to the left renal hilum

**Figure 2:**
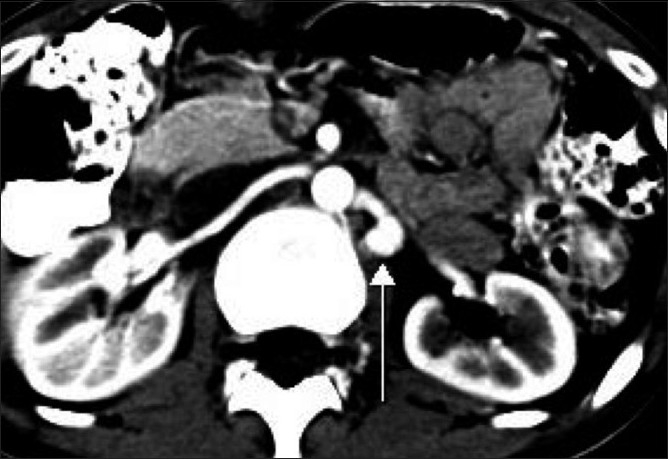
Axial contrast-enhanced CT scan shows homogeneous contrast enhancement within the structure that was overlapping the left renal artery

**Figure 3 (A-D):**
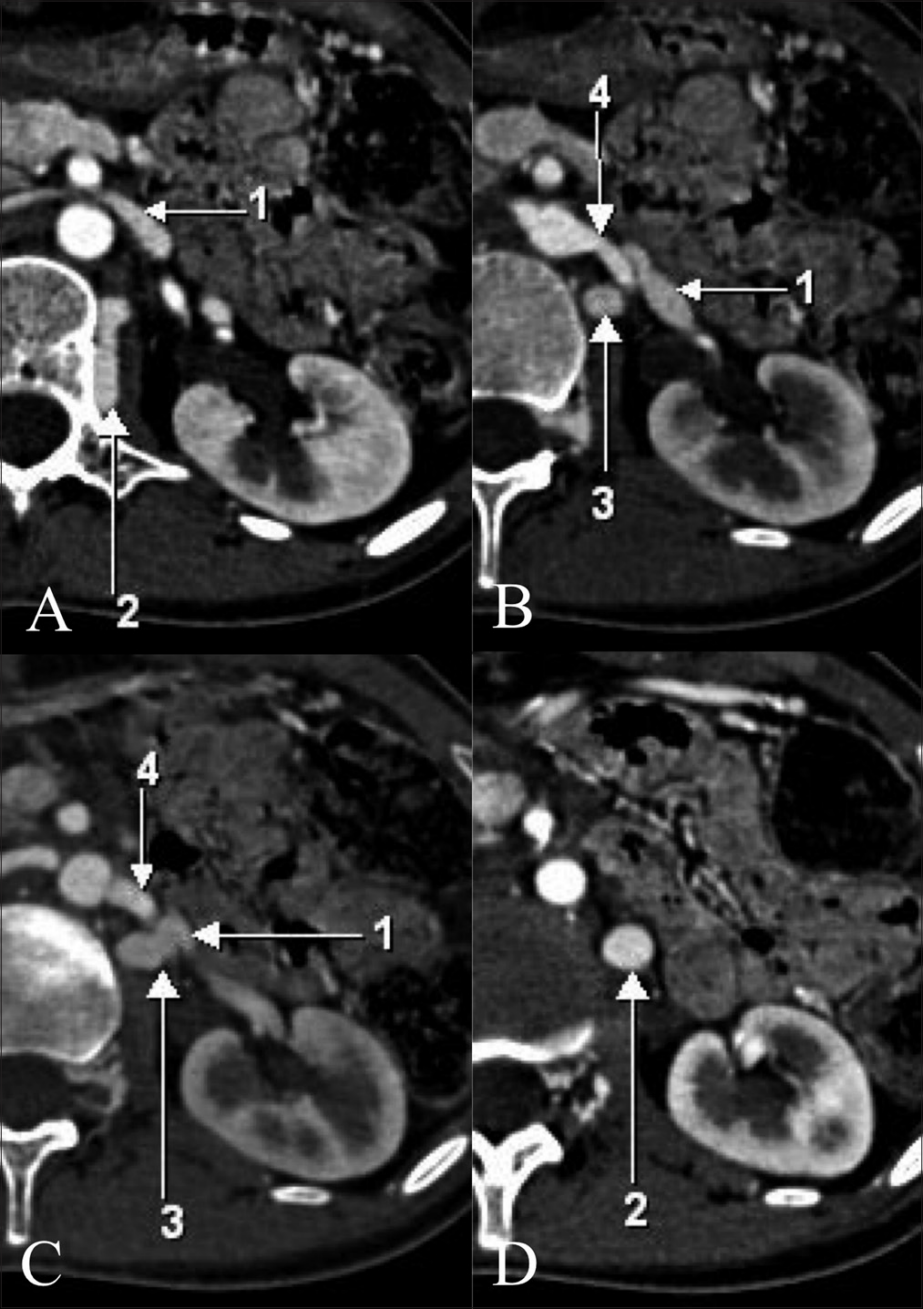
Narrow collimation, sequential, axial contrast-enhanced CT scans showing the relationships of the various vessels. Arrow 1 - left renal vein, arrow 2 - communicating vein between the left renal vein and the left ascending renal vein, arrow 3 - left ascending lumbar vein, arrow 4 - left renal artery

**Figure 4:**
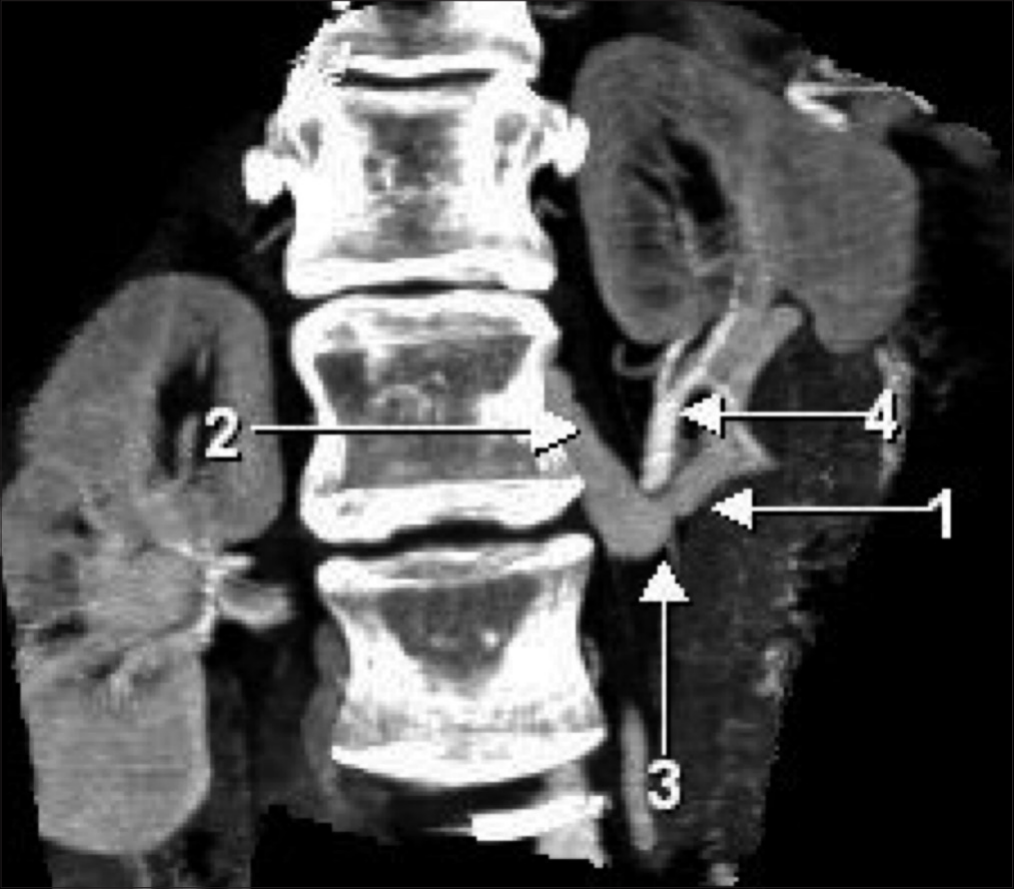
Coronal reformatted maximum intensity projection CT scan shows the varicosity of the communicating vein between the left renal vein and the left ascending lumbar vein in close proximity to the left renal artery. (The arrows point to the same structures as in Figure 3)

**Figure 5:**
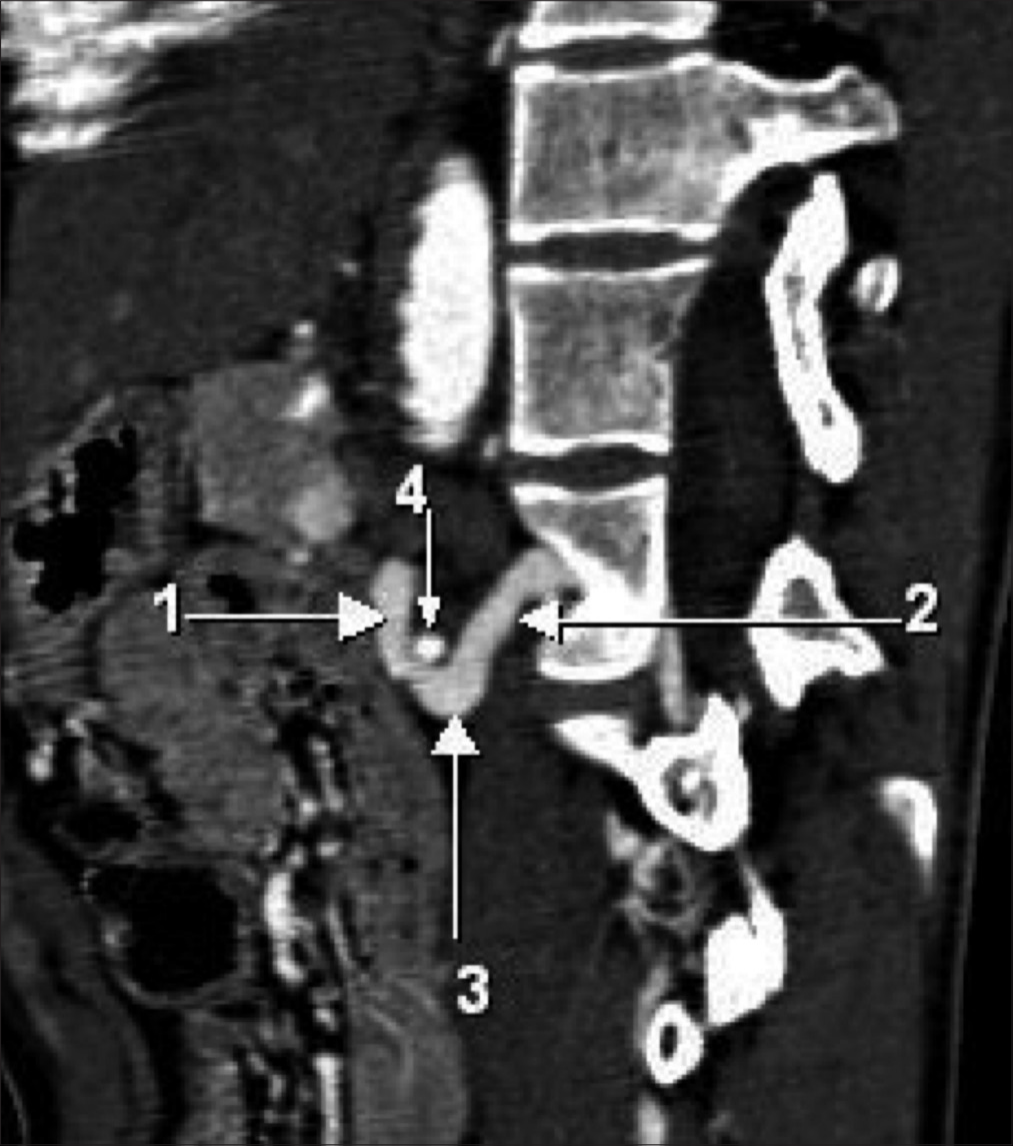
Sagittal reformatted maximum intensity projection CT scan shows the varicosity of the communicating vein between the left renal vein and the left ascending lumbar vein straddling the left renal artery. (The arrows point to the same structures as in Figure 3)

**Figure 6:**
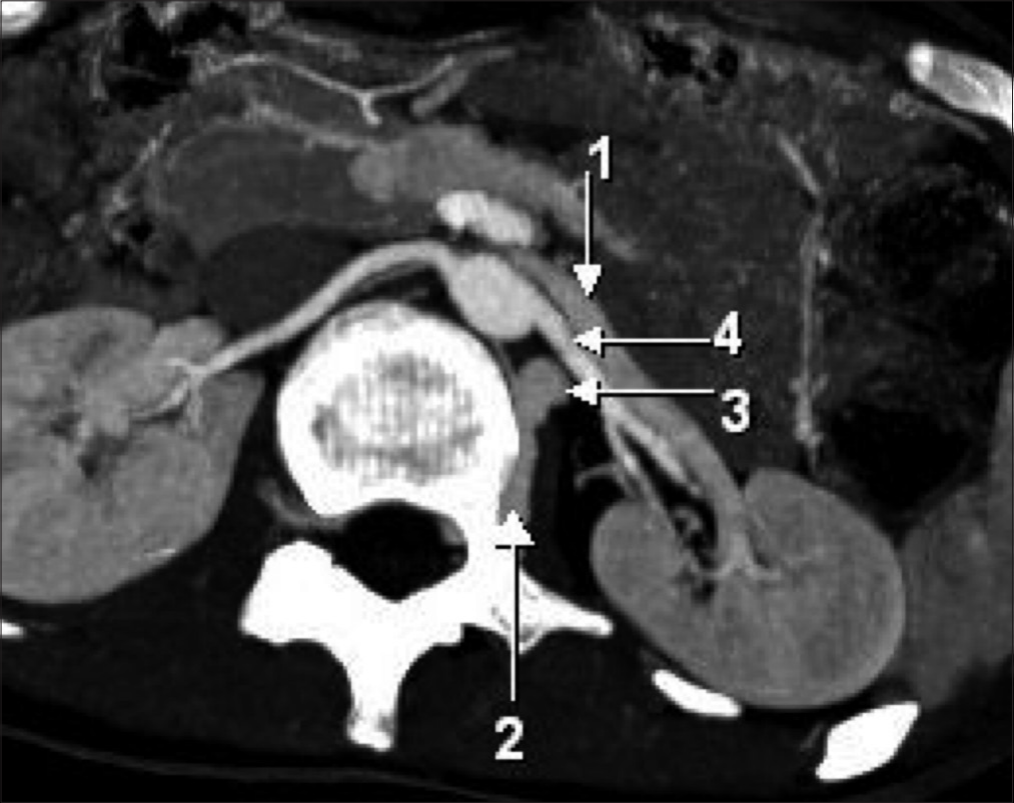
Axial maximum intensity projection CT scan shows the left renal vein, the left renal artery, and the communicating vein as distinct structures. (The arrows point to the same structures as in Figure 3)

A 19-year-old female presented with recurrent abdominal pain in the epigastric region associated with nausea and nonbilious vomiting for the past 1 year. She was treated symptomatically with antacids and pain killers, with temporary relief of symptoms. There was no significant past medical or surgical history. Physical examination and routine laboratory investigations were unremarkable. USG of the abdomen and pelvis was within normal limits. A CT scan of the abdomen (5-mm slice width/2.5-mm slice gap) demonstrated a well-defined isodense structure on the nonenhanced scan, adjacent to the left renal hilum [Figure 1], showing homogeneous enhancement [Figure 2]. This structure was seen overlapping the left renal artery and was presumed to be a renal artery saccular aneurysm. A repeat CT scan was performed with a narrow collimation (slice width: 1 mm/slice gap: 1 mm) for assessment of the left renal artery and vein [Figure 3], which revealed a varicosity of the communicating vein between the left renal vein (LRV) and the left ascending lumbar vein (LALV) [Figure 3]. This was located in close proximity to the left renal artery, but was distinctly separate from it. This was confirmed on multiplanar reformatted images [Figures 4–6], which revealed this varicosity to be straddling the left renal artery. The patient was managed conservatively.

## Discussion

Varicose veins are enlarged and tortuous venous channels commonly caused by retrograde flow due to incompetency of valves or obstruction to flow.[5] This term commonly refers to the veins of the lower extremities, although varices can occur at any site in the body, e.g. esophagus, stomach, intestines, scrotum, vulva, ovary, kidneys, and the retroperitoneum.[6–9] A communicating vein between the LRV and the LALV is a rare anomaly, and varicosity of such a communicating vein is extremely rare, with sparse reports in the literature. A normal communicating vein between the LRV and the LALV, or an azygous system, have been reported in 69-91.5% of the autopsy studies.[10] On phlebographic studies, the incidence of this normal venous communication has been variably found to be between 34 and 75%,[11,12] whereas the incidence on CT scan is around 35%. The disparity between the radiologic and anatomic incidence of this venous channel may be attributed to the limited resolution of the CT scan images.[13] Varicosity of this venous communication has been reported in 9% of the patients on phlebographic studies.[11] The first case report of varicosity of a venous communication between the LRV and the LALV demonstrated on CT scan was by Lien *et al*.,[1] and anecdotal reports have appeared since, describing similar cases.[13]

Dilated retroperitoneal venous channels have been confused with adrenal masses,[2] paraaortic lymph nodes,[14] and renal artery aneurysms. The identification of this varicosity as a normal variant is important as it can mimic normal and pathological entities that are located in close proximity to the left renal hilum and left paravertebral region. Awareness of varicosity of this communicating vein is of utmost importance for surgeons performing aortoiliac surgeries and nephrectomies as it may pose technical difficulties during surgery or cause life-threatening retroperitoneal hemorrhage.[3,4] On CT scan, recognition that this entity is of vascular origin and not an enhancing enlarged paraaortic lymph node is important in patients with testicular tumors as these malignancies tend to disseminate to the paraaortic lymph nodes via the lymphatics.[14]

With advances in multidetector computed tomography (MDCT) scanners, smaller, vascular structures can be easily imaged with a narrow collimation and contrast enhancement. The enhancement pattern of this varicosity and its contiguity on consecutive sections is usually sufficient to differentiate it from other nonvascular structures in the paraaortic and left renal hilar region; however, it should be noted that with the reduced scanning times on MDCT scans,the CT scan study is often completed even before the contrast can sufficiently opacify this communicating vein.[13]

A definite cause for varicosity of this vein has not been discussed earlier in the literature. However, a mechanism of valvular incompetence similar to that seen in the peripheral veins may be the predisposing factor. Varicosity of this vein may also be caused due to backpressure changes in the renal vein caused by various obstructive lesions, e.g. renal vein thrombosis or postoperative renal vein ligation.[15] The nutcracker phenomenon is characterized by compression of the LRV between the abdominal aorta and the overlying superior mesenteric artery due to reduction of the aortomesenteric angle.[16] Compression of the renal vein due to this phenomenon may cause an increase in the left renal venous pressure, which may result in varicosity of the communicating vein between the LRV and the LALV.

Our patient presented with abdominal pain, and no significant abnormality was seen other than a varicosity of this venous channel. The association between anomalous vessels and gastrointestinal complaints is complex and completely conjectural at this point of time. The left lumbar nerve plexus lies in close relation to this venous channel in the left paravertebral region. Dilatation of this vessel may cause compression and irritation of the left lumbar nerve plexus, which in turn may cause abdominal pain and nausea. A similar mechanism, wherein dilated venous channels cause abdominal pain due to nerve irritation, has been proposed with the nutcracker phenomenon[17] and in cases where inferior vena caval obstruction causes dilatation of the epidural venous plexus and radiculopathy.[18]

In conclusion, varicosity of the communicating vein between the LRV and the LALV is a rare entity with sparse literature about its imaging appearances. Nonetheless, its recognition is important during presurgical evaluation in renal transplant donors and in those undergoing aortoiliac surgeries. This entity may be commonly confused with paraaortic lymphadenopathy, adrenal pseudo-masses, and aneurysms of the renal vasculature. The proposed hypothesis for the etiology of the abdominal pain and nausea is purely conjectural and needs validation.
